# Phosphorus-Induced Adaptation Mechanisms of Rye Grown on Post-Flotation Copper Tailings

**DOI:** 10.3390/biology10080818

**Published:** 2021-08-23

**Authors:** Piotr Stępień, Krzysztof Gediga, Zofia Spiak

**Affiliations:** Department of Plant Nutrition, Wroclaw University of Environmental and Life Sciences, ul. Grunwaldzka 53, 50-357 Wroclaw, Poland; krzysztof.gediga@upwr.edu.pl (K.G.); zofia.spiak@upwr.edu.pl (Z.S.)

**Keywords:** copper tailings, H^+^-ATPase, low-molecular-weight organic acids, phosphorus availability, photosynthesis, revitalisation, rye

## Abstract

**Simple Summary:**

The mining activities for the production of copper, lead, zinc, and others are inevitably associated with the generation of an enormous volume of waste materials, i.e., tailings. The global area covered with tailings is on the order of hundreds of millions of hectares, with this being predicted to rise significantly in the coming decades. Importantly, the physicochemical properties of tailings, such as high content of heavy metals and high pH, pose a serious threat to the surrounding ecological environment. This, combined with low available nutrients, makes revegetation of degraded lands very difficult, with the number of field trials demonstrating successful revitalisation remaining very low. In this study, we investigate in rye, as a model plant, a wide array of physiological processes and their significance in determining survival on the copper tailings. We demonstrate that limitations in plant growth on such wastes is not simply related to high copper content. Rather, we present data that the low availability of phosphorus and activity of the mechanisms involved in phosphorus extraction from the rhizosphere are important determinants of the plant growth and survival rate. With these results, we make a direct and significant contribution towards meeting future demands for effective revitalisation techniques of degraded lands.

**Abstract:**

Although a considerable effort has been made over the last decades to develop cost-effective phytotechnologies as an alternative to conventional techniques for the management of contaminated lands, successful revegetation of the tailings still represents a major challenge. Here, we evaluate the potential of rye (*Secale cereale* L.) for growth and survival on the tailings after copper (Cu) ore processing. Four rye varieties were cultivated in a pot experiment on the post-flotation sediment with increasing phosphorus (P) doses (22, 44, 66, 88, and 110 mg·kg^−1^). The resistance of the studied rye genotypes to stress was assessed by observing the growth and development of plants, determining the dry mass accumulation, the Cu and P uptake and content, and a number of physiological parameters related mainly to P mobilisation. Exposure of tested rye varieties to high Cu concentrations in the tailings did not result in any significant plant mortality, with the intracellular Cu concentrations being below the critical toxic level. In contrast, the low availability of P due to alkaline properties of the tailings and the mechanisms involved in the mobilisation of sparingly soluble forms of this element (i.e., H^+^-ATPase-driven proton efflux in roots and organic acid exudation), were identified as main factor determining the level of tolerance. The efficiency of the photosynthetic activity was a key determinant for the P-mobilising capacity of rye. We further showed that rye varieties with more primitive genetic background might be potentially more suitable for growth on the post-flotation copper tailings. The results provide important and novel knowledge that will certainly support future works in developing strategies for successful revitalisation of degraded lands.

## 1. Introduction

The dynamic industrial development during the last few decades has resulted in an intense global exploitation of metallic resources. Copper (Cu), zinc (Zn), and lead (Pb) are still among the most widely used in society, and, at present, there are no really good substitutes available for these metals, considering their specific technical and chemical properties [1]. The production of Cu, Pb, Zn, and others, however, is inevitably associated with the generation of an enormous volume of waste materials, i.e., tailings. Tailings, defined as a mixture of crushed rocks and processing fluids, have been described in detail [2,3,4,5]. They constitute 94–96% of processed ore, and extraction of the lower-grade ores has become a long-term trend, contributing to an increased production of the waste materials [6,7]. The global area covered with tailings is on the order of 100 million hectares, containing several hundred thousand million tonnes of mine waste, with another 20,000–25,000 Mt added to the piles every year [8]. The physicochemical properties of tailings, such as high content of heavy metals, high amounts of very fine silt and clay particles, and high pH, pose a serious threat to the surrounding ecological environment [9,10,11]. Those characteristics, combined with unsuitable air–water conditions, the low available nutrients (with phosphorus (P) being least available element), and almost no organic matter in the waste materials, are factors limiting the potential for successful revegetation of tailings [4,12,13,14].

Many methods have been applied for the rehabilitation of degraded lands, including covering tailings with nonpolluted materials to reduce environmental hazards. This, however, is expensive and impractical due to the large areas occupied by the waste products and the huge cost of the soil or organic matter transportation [15]. It is now widely accepted that stabilisation of tailings by vegetation is far more desirable than the above method. According to this concept, native or cultivated plant species with a high level of tolerance to the adverse conditions are used for rehabilitation [16,17,18]. Among a number of plants, Fabaceae species, e.g., white sweet clover (*Melilotus albus*), alfalfa (*Medicago sativa*), or red clover (*Trifolium pratense*), meet these criteria to a high extent. They not only offer the advantage of enriching the poor substrate in nitrogen (N) due to symbiosis with *Rhizobium* bacteria, but also produce a large amount of biomass, contributing to an increase in the soil organic matter, and develop an extended root system, improving the physical properties of the substrate [19,20]. Mixtures of Fabaceae plants and grasses are often used in revegetation due to the possibility of creating a compact turf covering the waste materials, thus effectively reducing surface erosion and the dust lift-off from the tailing dumps [21,22]. Although cereals are rarely used individually during the biological rehabilitation of the post-industrial areas, they are proposed to be cultivated in the crop rotation systems specifically developed for reclamation. During the experiments carried out so far, the use of wheat, barley, and oat has been mainly reported [23,24]; however, rye varieties are increasingly being used for this purpose [25,26]. Rye withstands adverse conditions significantly better than other cereals. A characteristic feature of rye is high tolerance to extreme soil pH, low N and P availability, and the presence of aluminium and manganese ions in the rhizosphere. Importantly, its resistance to both drought and waterlogging enables it to produce a soil-binding cover on land, where other cereals will not grow. Therefore, regardless of the ground type, the differentiation of the crop yield is low for rye varieties [27,28].

A considerable effort has been made during the last few decades to develop cost-effective in situ phytotechnologies as an alternative to conventional techniques for sustainable remediation and management of contaminated lands [13,29,30]. Studies so far reported have focused on screening of the tolerant plant species and genotypes for application in phytoremediation [31]. However, the contribution of the mechanisms functioning at the level of plant cells to determining high stress tolerance and supporting revegetation of degraded lands has not been widely investigated. Importantly, the number of field trials demonstrating successful remediation remains well below the total number of studies carried out.

The aim of the study was to evaluate the potential of rye varieties for growth on the post-flotation copper tailings. Previously, the authors successfully demonstrated the cultivation of Fabaceae plants [22]. They were awarded with a gold medal at the Belgian and International Trade Fair for Technological Innovation “Innova 2012”, Brussel. Here, we investigated in rye, as a model plant, the extent to which some physiological processes are induced under such conditions, and how their capacity determines plants survival on metalliferous wastes.

## 2. Materials and Methods

### 2.1. Properties of the Sediment

During the experiment, the post-flotation sediment after copper ore processing collected from the tailing pond located in Iwiny (51°12′38.34″ N, 15°40′53.58″ E), Lower Silesia province, Poland, was used as a substrate. The total amounts of some elements (Table 1) in the sediment were determined by ICP-AES after microwave digestion using a MARS 6 system (CEM, Matthews, NC, USA) in concentrated HNO_3_ and H_2_O_2_. The post-flotation material contained very high amounts of calcium and magnesium (mainly as carbonates and silicates), a relatively high total content of P, and an extremely high content of Cu.

Due to the high abundance of carbonate minerals, the sediment was characterised by an alkaline pH value ranging between 8.2 and 8.5. The available forms of magnesium determined according to [32] and potassium according to [33] were relatively high—159 and 194 mg·kg^−1^, respectively. The available Cu concentration in the post-flotation sediment determined in the water extract (1:10) and extracted with 0.01 M CaCl_2_ was 0.052 mg·kg^−1^ and 0.36 mg·kg^−1^, respectively. The Rhiem DL available P was only 5.5 mg·kg^−1^. The deposited material consisted mainly of the fractions with a diameter below 0.05 mm due to fine grinding of the copper ore (Table 2).

### 2.2. Plant Material and Treatment

Seedlings of four Polish rye (*Secale cereale* L.) varieties were cultivated in a pot experiment. Among the genotypes studied, Horyzo, Domir, and Dankowskie Rubin, belong to modern, intensive varieties, whereas Dankowskie Zlote is an old, extensive variety, widely cultivated over the past six decades [27]. The plants were grown under a 16 h photoperiod (PPFD 300 μmol·m^2^·s^−1^) at 23/19 °C (day/night). The relative humidity in the growth chamber was maintained at 70%. The 0.5 L PVC pots (12 cm height, 15 cm diameter) were filled with mixture of 450 g of sediment and 50 g of washed sand. Seeds of rye varieties were sown into the pots, and 20 uniformly sized seedlings were left after thinning. Plants were supplemented with 5 mL of Ca(NO_3_)_2_ solution containing 10 mg N·mL^−1^ and 0, 5, 10, 15, 20, and 25 mL of Ca(H_2_PO_4_)_2_·H_2_O solution containing 2.2 mg P·mL^−1^, with the resulting final amounts of P incorporated being as follows: 0 (0P), 22 (1P), 44 (2P), 66 (3P), 88 (4P), and 110 (5P) mg·kg^−1^. Pot positions were randomised every week and watered daily to 60% of the field capacity as measured by weight. Plants were harvested 28 days after sowing at BBCH stage 14 (four leaves) according to [34] and freshly weighed (FM). The fresh root tissue was collected, and the shoots were dried at 70 °C for 72 h and reweighed for determination of dry mass (DM). The samples were used for the subsequent analytical procedures.

### 2.3. Gas Exchange and Chlorophyll Fluorescence

Gas exchange parameters were monitored using an Infrared Gas Analyser (IRGA) CIRAS-1 (PPsystems, Hitchin, Hertfordshire, UK) provided with a *PLC-B Parkinson* Leaf Chamber (2.5 cm^2^). The assimilation rate (*A*; μmol·m^2^·s^−1^), stomatal conductance (*g_s_*; mmol·m^−2^·s^−1^), transpiration rate (*E*; μmol·m^2^·s^−1^), and internal CO_2_ concentration (*C_i_*; μmol·mol^−1^) were measured on intact leaves at a PPFD of 850 μmol·m^2^·s^−1^ after 30 min to achieve steady-state conditions. Leaf temperature within the growth chamber was controlled at 25 ± 1 °C, and the CO_2_ concentration supplied was 415 μmol·mol^−1^.

Chlorophyll fluorescence was measured using a pulse-modulated amplitude fluorometer FMS-2 (Hansatech Instruments Ltd., Pentney, Norfolk, UK) at first in the leaves overnight adapted to darkness and then in those exposed to an actinic light of 850 μmol·m^2^·s^−1^. Saturating pulses of white light (9500 μmol·m^2^·s^−1^) were supplied to obtain Fm and Fm’ values. The ΦPSII, Fv/Fm, and NPQ parameters were calculated as previously described [35].

### 2.4. Purification of Plasma Membrane

Plasma membrane (PM) vesicles were isolated from the rye roots. The plant material (fresh root tissue) was homogenised in ice-cold 50 mM MOPS–KOH buffer (pH 7.5), containing 330 mM sorbitol, 5 mM EDTA (ethylenediaminetetraacetic acid), 5 mM DTT (dithiothreitol), 5 mM ascorbate, 0.5% (*w/v*) PVPP (polyvinylpolypyrrolidone), and protease inhibitor cocktail (Thermo Scientific, Waltham, MA, USA). The homogenate was passed through a 200 µm SpectraMesh filter (Spectrum Labs, Rancho Dominguez, CA), centrifuged at 10,000× *g* for 15 min, and the resulting supernatant was centrifuged at 45,000× *g* for 30 min. The microsomal pellet was resuspended in 10 mM potassium phosphate buffer (pH 7.8), containing 330 mM sorbitol, 0.5 mM EDTA, and 1 mM DTT, and purified by phase partitioning according to [36]. The suspension was applied to an aqueous polymer two-phase system consisting of 6.2% (*w/w*) Dextran T500, 6.2% (*w/w*) PEG 3350, 330 mM sorbitol, 10 mM potassium phosphate (pH 7.8), and 5 mM KCl. The final upper phase was pelleted by centrifugation at 100,000× *g* for 1 h and resuspended in 10 mM potassium phosphate buffer (pH 7.8), containing 330 mM sorbitol, 0.5 mM EDTA, 1 mM DTT, and 5 mM KCl. The PM obtained via this procedure was well purified, composed mainly of the right-side-out vesicles, which were used for determining the hydrolytic ATPase activity. Some of the PM vesicles were turned inside-out by treatment with Brij-58 according to [37] and used for the measurements of ATP-dependent H^+^ transport across the PM. Protein content was determined using a Bradford protein assay kit (Sigma-Aldrich, St. Louis, MO, USA), according to the manufacturer’s instructions.

### 2.5. PM H^+^-ATPase Hydrolytic Activity and H^+^ Transport

The hydrolytic activity of the vanadate-sensitive ATPase was determined according to [38], modified by [39]. The reaction mixture contained 50 μg of PM protein, 33 mM Tris–Mes (pH 7.5), 3 mM ATP, 2.5 mM MgSO_4_, 50 mM KCl, 1 mM NaN_3_, 0.1 mM Na_2_MoO_4_, 50 mM NaNO_3_, 0.02% Triton X-100, and 200 μM Na_3_VO_4_. PM H^+^-ATPase activity was determined from the difference between the activity measured in the absence and the presence of Na_3_VO_4_. The P_i_ released in the reaction was assayed according to [40] with 0.2% (*w/v*) SDS being applied to prevent precipitation.

The activity of H^+^ transport across PM was measured spectrophotometrically as the change in acridine orange absorbance at 495 nm (A_495_) according to [41] with slight modifications. The assay medium contained the PM vesicles (50 μg protein), 20 mM MOPS–KOH (pH 7.5), 330 mM sorbitol, 40 mM KCl, 0.1% BSA, 10 μM acridine orange, and 0.1% Brij-58. Proton transport was initiated with 3 mM Mg-ATP added to the reaction mix. The correction for the passive proton movement through the PM was determined without ATP in the assay medium.

### 2.6. Determination of ATP

The fresh root tissue pre-ground in liquid nitrogen to a fine powder was homogenised in 4.5% perchloric acid (PCA). The mixture was briefly vortexed and incubated on ice for 5 min, followed by centrifugation at 15,000× *g* for 2 min. The excess PCA in supernatant was precipitated with 2 M ice-cold KOH, with the pH being adjusted to 7.75, and the supernatant was re-centrifuged at 15,000× *g* for 10 min. ATP was determined fluorometrically by glycerol phosphorylation (λ_ex_ = 535 nm, λ_em_ = 587 nm) using the Colorimetric/Fluorometric Assay Kit (Sigma-Aldrich).

### 2.7. Determination of Organic Acids and P in Root Exudates

The MicroRhizon lysimeter microsamplers (Rhizosphere Research Products, Wageningen, The Netherlands) for soil solution collection were maintained in each pot at half of the substrate height. The solutions collected from the rhizosphere were analysed for inorganic phosphate (Pi), determined using the ammonium molybdate colorimetry method at 714 nm [42,43] with an Evolution 600 UV/Vis spectrophotometer in cuvettes with 10 mm path length (Thermo Scientific, Waltham, MA, USA). Subsamples of the MicroRhizon collection were filtered through a 0.22 μm Millex GP syringe filter (Millipore Ireland, Tullagreen Carrigtwohill, Cork, Ireland). The filter-sterilised root exudates were evaporated to dryness using a Savant™ SpeedVac™ concentrator (Thermo-Fisher), resolved in 200 μL of MilliQ-grade water and stored at −80 °C. Low-molecular-weight organic acids (OAs) present in the concentrated root exudates were determined by HPLC, injecting the samples (40 μL) onto a ZORBAX Eclipse C18 column (250 mm × 4.6 mm; Agilent, Santa Clara, CA, USA) maintained in a thermostated column oven. The HPLC mobile phase, containing degassed 5 mM H_2_SO_4_ solution in MilliQ-grade water and absolute methanol (95:5), was delivered at a flow rate of 0.4 mL·min^−1^. The eluted OA were detected at 210 nm with a diode-array detector and quantified by a comparison of the peak surface areas with values obtained for the following standards: acetic, citric, malic, and oxalic acids.

### 2.8. Elemental Analyses

The dried plant material was ground in an IKA MF 10 stainless-steel mill (IKA^®^-Werke GmbH & Co. KG, Staufen, Germany). The concentration of Cu was determined using the atomic absorption spectroscopy (AAS) method with a SpectrAA 220FS spectrometer (Varian, Melbourne, Australia) following dry mineralisation in a muffle furnace at 450 °C and dissolving the ash in 1 mol·dm^−3^ nitric acid. The P concentration in plant material was determined using the ammonium molybdate colorimetry method at 714 nm [42,43]. The values of Cu and P uptake were calculated by multiplying the Cu and P concentration, respectively, by the dry mass yield.

### 2.9. Statistical Analyses

To examine the treatment effects, i.e., varieties and P dose, on shoot biomass, P and Cu concentration and uptake, the OA and P concentration in soil solutions, and the physiological parameters, data were subjected to two-way ANOVA after verification of normal distribution by Shapiro–Wilk’s test and data Box-Cox transformation. Statistics were calculated on the basis of *n* = 9 replications. Error bars in all figures represent 95% confidence interval (interaction P dose × variety) after post hoc Tukey HSD test, with the letters denoting homogeneous groups being omitted for a clear view. All statistical analyses were performed with Statistica software version 13 (TIBCO Software Inc., Palo Alto, CA, USA, 2017). The regression analyses for the relationships between inorganic P in the soil solutions and the P concentration in shoots, the H^+^-ATPase-driven proton efflux, and the OA exudation were conducted with QtiPlot 1.0.0-rc13 software (release date 11 June 2020, Copyright 2004–2020 IonVasiliev) at a significance of *p* < 0.05.

## 3. Results and Discussion

### 3.1. The High Cu Content Is Not the Most Critical Factor Limiting Revegetation of the Post-Flotation Tailings; It Is Low Bioavailability of P

**Figure 1:**
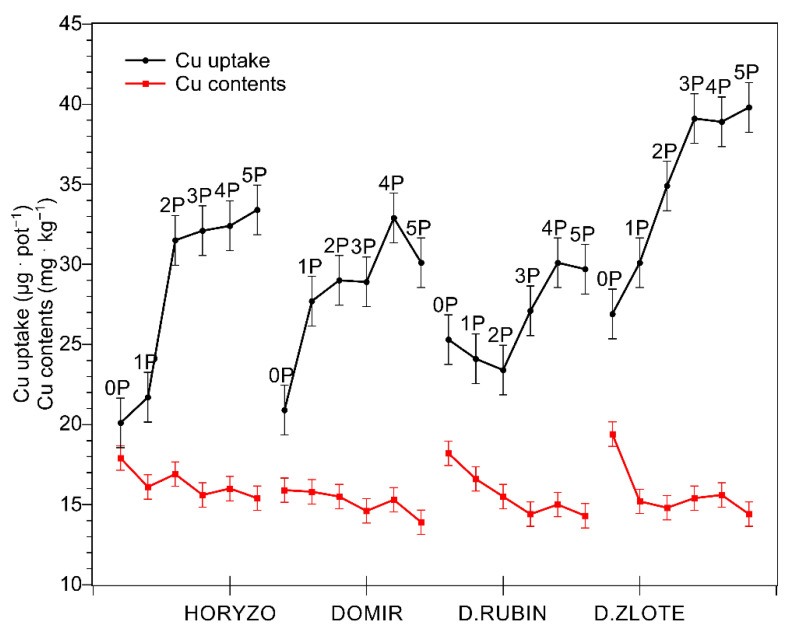
Changes in the copper (Cu) uptake and concentration in shoots of rye varieties grown on the post-flotation sediment. Plants were supplemented with 0 (0P), 22 (1P), 44 (2P), 66 (3P), 88 (4P), and 110 (5P) mg P·kg^−1^. The shoot tissue samples were collected 28 DAS at the BBCH 12 stage, dried, and used for determining Cu concentrations. Data represent the means of *n* = 9 replications, and vertical bars represent 95% confidence intervals after a post hoc Tukey HSD test (interaction P dose × variety), with the letters denoting homogeneous groups being omitted for a clear view.

**Figure 2:**
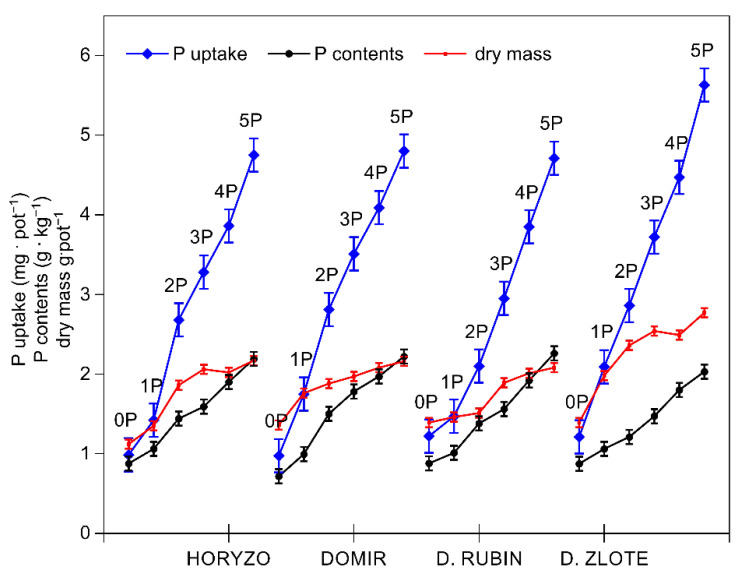
Changes in the phosphorus (P) uptake and concentration, and the dry mass accumulation in shoots of rye varieties grown on the post-flotation sediment. Plants were supplemented with 0 (0P), 22 (1P), 44 (2P), 66 (3P), 88 (4P), and 110 (5P) mg P·kg^−1^. The shoot tissue samples were collected 28 DAS at the BBCH 12 stage, dried, and used to determine P concentrations and dry mass. Data represent the means of *n* = 9 replications, and vertical bars represent 95% confidence interval after a post hoc Tukey HSD test (interaction P dose × variety), with the letters denoting homogeneous groups being omitted for a clear view.

In spite of natural succession lasting over two decades, the surface of the investigated tailing pond remained almost bare, with only very poor vegetation [44]. Among the trace elements determined in the course of the experiment, Cu was the main contaminant present in the investigated post-flotation tailings. Although the mined ore is being strongly depleted of Cu during its processing, the total content of this element measured in the tailings was as high as 0.18% of dry mass, i.e., 1800 mg·kg^−1^ (Table 1). Such extreme values exceed the maximum permissible level of Cu in European agricultural soils by more than 12 times [45]. Within this range of concentrations, the inhibition of metabolism and high lethality of plant organisms may be expected [46]. However, despite such unfavourable conditions for vegetation, we observed rather undisturbed growth of tested plants on the post-flotation sediment during the current experiment. Exposure of four rye varieties to the high Cu concentrations did not result in significant mortality. The uptake of Cu increased sharply in shoots of all varieties (Figure 1) with increased dry mass accumulation (Figure 2) resulting from P supplementation. Cu uptake was most prominent in Dankowskie Zlote, which, however, demonstrated concentrations of this metal that did not differ significantly from those measured in other rye varieties, probably due to an intense build-up of dry mass and the dilution effect. The biological response and the metabolic burden imposed on plants are a function of the abundance of toxic ions at a cellular level [35]. Cu concentrations determined in the control leaf tissue ranged from 16 to 19.5 mg·kg^−1^ dry mass (Figure 1). Increasing amounts of P resulted in a progressive decline in the intracellular Cu content, with the lowest Cu levels being generally found at the highest P dose of 110 mg·kg^−1^ (5P). Thus, the Cu concentration observed in tested rye varieties grown on the post-flotation sediment were within the range normally found in plants (5–20 mg·kg^−1^ [47]) and only slightly above the range of expected concentrations, i.e., 5–15 mg·kg^−1^ [48,49]. Importantly, the obtained values were below the commonly accepted range of critical toxic level for plants (20–100 mg·kg^−1^; [45]), which rather excludes the possibility of Cu toxicity incidents at the cellular level.

The relatively low Cu concentrations detected in the green tissue of rye varieties might be explained in a number of ways. Most simply, this could reflect an efficient Cu-excluding mechanism functioning in rye. Gramineous species are generally regarded as metal excluders, i.e., plants which effectively limit heavy-metal translocation and maintain relatively low levels in their shoots over a wide range of soils [46]. The results obtained here are in line with our recent studies on the chemical composition of a number of native plant species colonising the post-flotation tailings, where the studied grass species accumulated significantly lower amounts of Cu than the examined species of dicotyledons [44]. The highest shoot Cu concentrations found in the latter reached nearly 50 mg·kg^−1^, with these values being almost 10 times higher than those determined in the reference plants grown on soil and being within the range of critical toxic Cu level for plants [45]. This is further supported by data available from our previous report where oat plants, another crop of the Gramineae family, cultivated on the same copper tailings demonstrated intracellular Cu concentrations not exceeding 18 mg·kg^−1^ [23].

In addition to the above, the limited Cu accumulation observed in rye varieties grown on the post-flotation tailings may be related, at least partially, to the physicochemical properties of the substrate itself, i.e., high pH and high abundance of carbonates. Soil carbonates have been shown to control the mobility and bioavailability of Cu in contaminated calcareous soils and in soils ameliorated with limestone [50]. The presence of free CaCO_3_ and MgCO_3_ generally reduces the solubility of trace elements, with carbonate/bicarbonate ions being involved in the formation of poorly soluble or insoluble metal carbonates. Due to limited Cu mobility attributed to the presence of carbonates in alkaline soils, the resulting Cu concentration in the soil solution is very low, comprising only 0.1–1% of the total Cu in soils [50,51]. Thus, the pH- and carbonate-mediated reduction in Cu availability in the soil solution may represent an indirect mechanism protecting the plants grown on the post-flotation tailings against Cu toxicity, in spite of the extremely high total content of this element in the sediment. Importantly, an additional reduction in the tissue Cu concentration arose from supplementation of the rye varieties with increasing amounts of P (Figure 1). P application into the post-flotation waste further decreased the accumulation of Cu in leaves of rye most probably due to the precipitation of Cu in soil solution as Cu_3_(PO_4_)_2_, characterised by extremely low solubility, K_sp_ = 1.4 × 10^−37^ [52]. In this context, the high total Cu contents found in the post-flotation sediment may not be, in fact, the most critical factor limiting the potential for effective revegetation of this type of waste.

The lack of nutrients or their low availability is an important selective factor, which hardly constrains vegetation development. Metalliferous wastes are invariably deficient in essential plant nutrients, particularly N and P [4,13]. P is a key limiting nutrient playing a vital role in determining the structures, functions, and processes of terrestrial ecosystems [53]. The common total content of this element in soils globally ranges from 0.02% to 0.08% D.M. [54]. The total P content determined in the studied copper tailings constituted 0.08% of dry matter (Table 1), which is higher than the averages in Polish soils, i.e., 0.042% [55], but lower than European mean values, 0.15% [56]. In spite of the high total P level found in the post-flotation sediment, the available P content determined by the Egner–Riehm DL method was only 5.5 mg·kg^−1^, with values below 22 mg·kg^−1^ being regarded as very low [57]. The low availability of this element may be attributed to the high pH and high abundance of CaCO_3_ and MgCO_3_ in the sediment. The excessive carbonates in alkaline conditions enhance P sorption on their surface, and the high concentrations of calcium and magnesium induce P precipitation in the form of poorly soluble Ca_3_(PO_4_)_2_ and Mg_3_(PO_4_)_2_, with K_sp_ values of 2.07 × 10^−33^ and 1.04 × 10^−24^, respectively [52]. This seriously restricts the bioavailability of P for plant organisms, exposing them to the threat of P starvation.

Indeed, the intracellular P concentrations determined in the control plants of all rye varieties were very low and ranged from 0.6 to 0.9 g·kg^−1^ dry mass (Figure 2). The control levels of P measured in rye during the present experiment are consistent with the P contents we previously reported in a range of native grass species colonising the investigated post-flotation tailings [44]. The typical P concentration in the aboveground parts of well-fed plants is in the range from 0.3% to 0.5% of dry matter during the vegetative stage of growth [58]. In small grain crops, including wheat, barley, oat, and rye, a P content of 0.15% is regarded as a critical value, at which a 5–10% yield reduction may be observed, and P contents of 0.2–0.5% are the optimal range [59]. The increasing amounts of P in the present experiment resulted in a rapid increase in P uptake in shoots of all varieties, with this effect being most prominent in Dankowskie Zlote and least defined in Dankowskie Rubin (Figure 2). The dry mass accumulation was induced with rising P uptake, most sharply in Dankowskie Zlote. The resultant intracellular P concentrations increased in leaves of all varieties from the control values to 2.3 g·kg^−1^ dry mass with the highest P dose being applied (Figure 2).

### 3.2. The Activity of the Mechanisms Involved in P Mobilisation in Rye Is Reversibly Correlated with P Availability in the Post-Flotation Tailings and Requires Initial P Supplementation

Plants have developed various strategies and a diverse array of morphological, physiological, and biochemical adaptations to overcome the low availability and poor mobility of soil P [60]. The exudation of OA and release of protons from the root system are among the most important mechanisms involved in the mobilisation of this element from sparingly soluble forms, especially in habitats with low P availability. Although the role of exudation has been extensively investigated in a number of species [61], the contribution of these mechanisms to P acquisition in rye and, in particular, their activity in plants grown on metalliferous wastes has not been studied.

The release of OA into the rhizosphere enhances P solubility, making it more available to plants [60,61]. Organic anions in carbonate sediments may displace P on adsorption sites on minerals and decrease the total amount of adsorption sites available. In addition, the organic anion may chelate metal and carbonate dissolution end products, such as Ca^2+^ ions, that would otherwise immobilise P [62].

**Figure 3:**
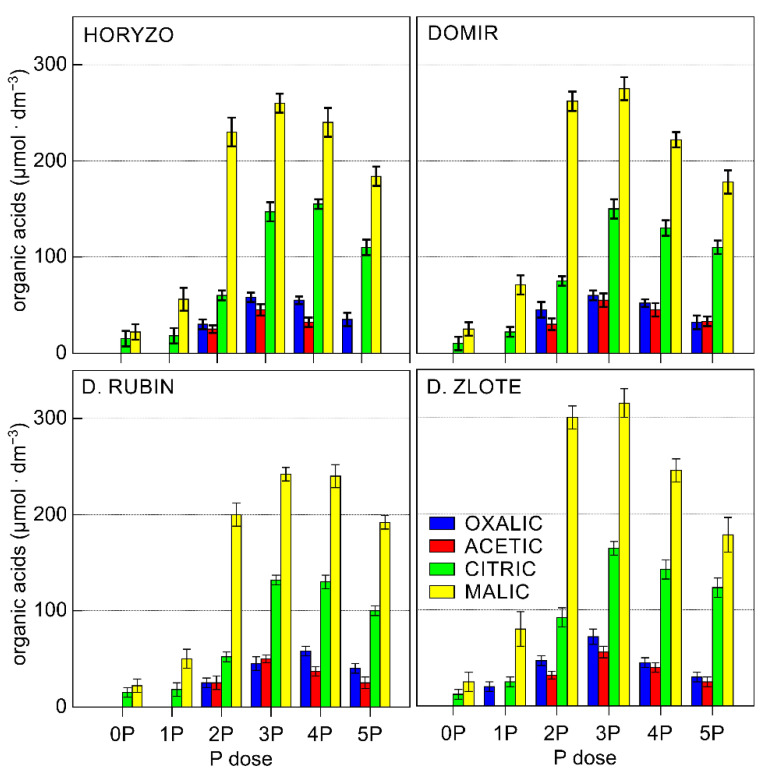
Effect of variable P supplementation on the organic acid (OA) exudation in roots of rye varieties grown on the post-flotation sediment. Soil solutions were collected directly from the root zone of intact plants with lysimeter microprobes, filter-sterilised, and analysed by UHPLC. Data represent the means of *n* = 9 replications, and vertical bars represent 95% confidence interval after a post hoc Tukey HSD test (interaction P dose × variety), with the letters denoting homogeneous groups being omitted for a clear view.

In the current study, the chromatographic analyses of the collected solutions resulted in the identification of significant amounts of four OAs: oxalic, acetic, citric, and malic acid. During the experiment, significant differences were observed in the exudation in four rye varieties (Figure 3). Among rye varieties studied, Dankowskie Zlote demonstrated the highest overall OA contents in the exudates derived from the rhizosphere. In contrast, the sum of OA determined in Dankowskie Rubin remained at the lowest level, irrespective of the level of P supply. In the case of Horyzo and Domir, the secretion of OA was characterised by intermediate values. The reported concentrations of particular organic anions measured in the soil solution usually ranged from 0.1 µM to more than 0.6 mM [63]. The sum of OA measured in the control plants (0P) of all rye varieties was very low, i.e., 37 μmol·dm^−3^ on average. This constituted 29–49% of the values recorded after applying the lowest P dose (i.e., 75–125 μmol·dm^−3^) and only 6–8% of the maximum exudation rates (465–615 μmol·dm^−3^). We suggest here that rye may adopt a distinctive, minimum intracellular threshold of P, below which the processes involved in the mobilisation of sparingly soluble forms of P in the rhizosphere are downregulated or inhibited. Thus, plants grown under severe P deficiency may be affected to the level that restricts their capacity to increase the exudation of OA in the root system. The results from our experiment are in line with previous findings in narrow-leaved lupin (*Lupinus angustifolius* L.), where all investigated genotypes grown under low P significantly enhanced the exudation of carboxylates, whereas no significant increase in exudation was observed under the very low P treatment [64]. We further propose that the factor limiting the secretory activity in rye varieties grown on the post-flotation sediment under severe P deficiency is the process of photosynthesis (discussed later in detail), which is a source of energy and organic forms of carbon. Root exudation clearly represents a significant carbon cost to plants, with young seedlings typically investing up to 40% of their photosynthetically fixed carbon in the root exudates [65].

The exudation rate determined in all rye varieties increased with the onset of P supplementation, with this being induced most rapidly in leaves of Dankowskie Zlote upon exposure to the 1P level and developed further to reach the maximum rate at the 3P dose (Figure 3). A further increase in P supplementation resulted in a reduction in the total amount of OA released in rhizosphere, implying a progressive deactivation of the mechanisms involved in the carboxylate exudation in response to increasing P availability by means of negative feedback. This is most likely due to optimisation of the energy and organic carbon consumption from photosynthetic processes, with the maximised balance between investments and returns being crucial for the growth and survival under adverse conditions [65].

The differences observed in the levels of exudation in roots of rye varieties were well correlated with the efficiency of P acquisition; Dankowskie Zlote, characterised by the highest secretion of carboxylic acids, absorbed more P from the post-flotation sediment than other varieties. In contrast, the lower exudation activity determined in Horyzo and Domir, as well as Dankowskie Zlote in particular, resulted in less effective mobilisation of P resources, as expressed by lower P uptake (Figure 2).

Different plant species exude different types of carboxylates, e.g., predominantly citrate and malate in white lupine, malonate in chickpea, malate in wheat, and a wide range of carboxylates in Proteaceae species [63,66,67]. In the current experiment, the predominant component of the exudates collected from the rhizosphere in all of the rye varieties was malic acid. The content of remaining carboxylates decreased according to the following sequence: citrate, oxalate, and acetate (Figure 3). The particularly high concentration of malate in the exudates of rye varieties seems surprising, given the highest effectiveness of citrate in the mobilisation of soil P. Citrate is more efficient than, in order, oxalate, malate/tartrate, acetate, and succinate/lactate at solubilising P [68]. This is most likely because the three carboxyl groups in citrate allow more effective complexing of metal ions than the dicarboxylic and monocarboxylic acids, with efficient release of adsorbed P from soil matrices [69].

The high abundance of malic acid observed in the rhizosphere of plants during the experiment might be explained in number of ways. Exudation of OAs, particularly citric and malic acids, is associated with a decrease in soil pH in response to P deficiency. Since OAs are released from the root cell predominantly in the anionic form, exudation of such anions requires the concomitant efflux of positively charged counter ions [60]. These are generally assumed to be protons provoking direct acidification of the rhizosphere, with the suggestion that malate may be of more significance in regulation of this process than citrate [69]. Such acidification is of particular importance in alkaline/calcareous soils, where P is rendered insoluble due to the sorption to Ca [70]. Thus, the intense exudation of malate in rye varieties grown on the post-flotation tailings could be attributed to the physicochemical properties of the sediment, i.e., high pH and high abundance of carbonates.

Furthermore, the production of malic acid is associated with a lower metabolic cost than citrate, engaging smaller amounts of organic carbon allocated from the process of photosynthesis. Moreover, malate can be directly produced in the reactions of dark fixation of CO_2_ in roots, catalysed by phosphoenolpyruvate carboxylase (PEPC) and malate dehydrogenase (MDH), which does not produce ATP and, thus, can continue under severe P deficiency when ADP and Pi are seriously limited [71]. This explains the highest proportion of malic acid in the rye exudates collected under the lowest P supply when the tissue concentration was very low (Figure 2); with increased level of P, the percentage of malate decreased in all rye varieties (Figure 3).

The root exudation of malic acid has also been identified as an important mechanism involved in the signalling and the root architecture responses to P deficiency, triggering inhibition of the primary root growth and lateral root proliferation [65]. The root architecture modifications in rye are essential in response to water and nutrient limitation, resulting in a more robust root system and higher tolerance to drought and low nutrient availability, in contrast to other cereals [72].

In addition to the OA exudation in roots, an important mechanism involved in increased mobilisation of the poorly available forms of P is protonation of the rhizosphere, resulting from the activity of the proton pump located in the root cell plasma membrane [73]. Using the chemical energy of ATP hydrolysis, the PM H^+^-ATPase extrudes protons from cells into the soil environment, promoting the transition of sparingly soluble phosphates into soluble compounds. This process is of particular importance in alkaline/calcareous soils [61].

**Figure 4:**
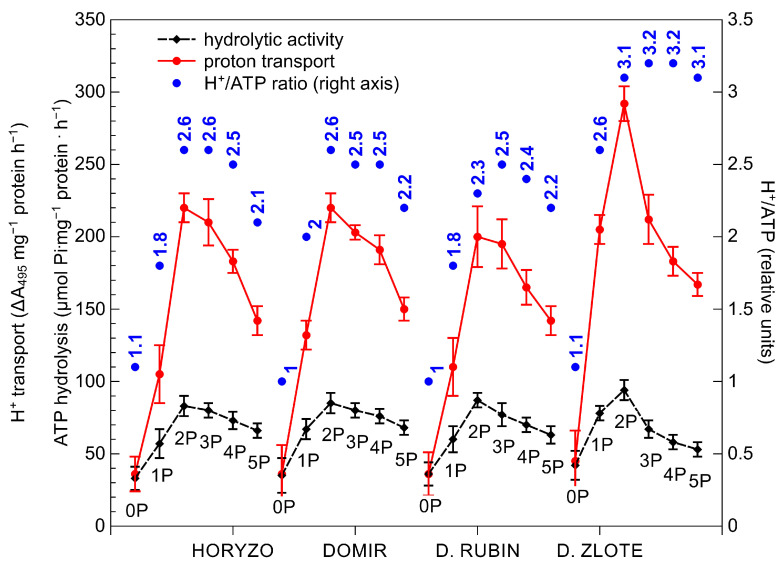
Effect of variable P supplementation on the H^+^-ATPase hydrolytic activity, the H^+^-ATPase-driven proton efflux, and the H^+^/ATP coupling ratio in roots of rye varieties grown on the post-flotation sediment. Plants were subjected to 0 (0P), 22 (1P), 44 (2P), 66 (3P), 88 (4P), and 110 (5P) mg P·kg^−1^. The plasma membrane vesicles were isolated from the root tissues, purified by two-phase partitioning, and the proton pump activities were determined spectrophotometrically. Data represent the means of *n* = 9 replications, and vertical bars represent 95% confidence interval after a post hoc Tukey HSD test (interaction P dose × variety), with the letters denoting homogeneous groups being omitted for a clear view.

The initiation of P supplementation resulted in a significant induction of the hydrolytic activity of H^+^-ATPase in roots of all rye varieties, most prominently in Dankowskie Zlote (Figure 4). The hydrolytic activity determined in this variety at its maximum, i.e., at the 2P dose, was around 15% higher than in the other varieties. Further supplementation with the 3P–5P doses resulted in a progressive reduction in the hydrolytic activity of H^+^-ATPase in all plants. The application of the lowest P dose provoked a rapid increase in the H^+^-ATPase-mediated proton transport through the root plasma membrane. This was induced most sharply in Dankowskie Zlote at the onset of supplementation to reach almost seven times the control level at the 2P dose. In comparison, the maximum intensities of H^+^ transport measured in Horyzo, Domir, and Dankowskie Rubin ranged from 5–5.5-fold of the initial rates and corresponded to the values achieved by Dankowskie Zlote already at the 1P dose. The application of the P doses higher than 44 mg·kg^−1^ (2P) resulted in a progressive reduction in proton transport across the plasma membrane in roots of all rye varieties, implying gradual suppression of the pumping activity of the plasma membrane H^+^-ATPase in response to increasing P availability. Furthermore, the H^+^/ATP coupling ratio determined in the plasma membranes was enhanced significantly upon exposure of the rye plants to the 1P level of supplementation (Figure 4). Among rye varieties, Dankowskie Zlote demonstrated in general the highest values of the H^+^/ATP coupling ratio across a range of P levels in the sediment.

An increased PM H^+^-ATPase activity induced in roots under P deficiency has been previously reported in various plant species, including white lupine, purple lupin, chickpea, and maize [61,73,74]. Changes in proton pump activity observed in plants during the current experiment may be due to various reasons. The activity of PM H^+^-ATPase is well known to be regulated by the 14-3-3 protein, with phosphorylation of the former polypeptide being a prerequisite for association of the latter. Binding of the 14-3-3 protein displaces the C autoinhibitory domain in PM H^+^-ATPase, resulting in its activation [75]. On the basis of results from our experiment, we propose that, in the control plants (0P), challenged with severe P deficiency, the intracellular Pi concentration is affected to a level that restricts enzymatic phosphorylation of the PM H^+^-ATPase polypeptide and, thus, the capacity to induce the proton release from the root system. On the contrary, as a result of the slightly increased Pi content at the onset of supplementation (1P; Figure 2), phosphorylation of the PM H^+^-ATPase and its interaction with the 14-3-3 protein are enhanced. This, in turn, provokes induction of the H^+^-ATPase activity and proton efflux across the plasma membrane, consistent with the role of the proton pump under P deficiency [74]. This conclusion is further corroborated by enhancement of the H^+^/ATP coupling ratio observed in our experiment in response to the initial increase of P supply (Figure 4). It has been shown that the formation of the 14-3-3/H^+^-ATPase protein complex leads to an enhanced H^+^/ATP coupling ratio of the PM H^+^-ATPase [76]. At the same time, the observed enhancement of the H^+^/ATP coupling ratio, most evident in Dankowskie Zlote, clearly indicates a more effective H^+^ transport per ATP unit. This is of high importance given the role of PM H^+^-ATPase in the mobilisation of insoluble P and energisation of the PHT1 phosphate transporters under P deficiency [68,77].

In addition to the above, the activity of the PM H^+^-ATPase plays an important role in the process of carboxylate exudation, due to the fact that the released protons provide a positive counter-charge to the secreted COOH anions [69]. Furthermore, the electrochemical potential created by H^+^-ATPase across the plasma membrane promotes the activity of the OA transporters (e.g., MATE/citrate transporters) and a passive, channel-mediated efflux of organic anions (e.g., ALMT1/malate channel) from the root [65]. Evidence has been provided that the chemical stimulation of the PM H^+^-ATPase activity with fusicoccin or its inhibition with vanadate resulted in the induction or reduction of the carboxylic acid exudation [74], with the efflux of malate being more tightly linked to proton release [60,78]. Indeed, the differences observed during our study in the H^+^-ATPase-mediated protonation of the rhizosphere (Figure 4) were clearly interrelated with the OA exudation (Figure 3). Dankowskie Zlote, demonstrating the highest exudation of carboxylates, was simultaneously characterised by the highest H^+^ release in the root zone. In contrast, the low proton transport rates determined in Dankowskie Rubin were associated with low levels of exudation. In Horyzo and Domir, both mechanisms involved in P mobilisation in the root system operated at intermediate levels. Importantly, the maximum activities of the PM H^+^-ATPase-driven proton transport in all rye varieties correlated well with the highest proportion of malic acid in the root exudates collected at the 1P–2P levels, consistent with the role of the malate-linked protonation of the rhizosphere in P mobilisation in alkaline/calcareous soils [69,70].

**Figure 5:**
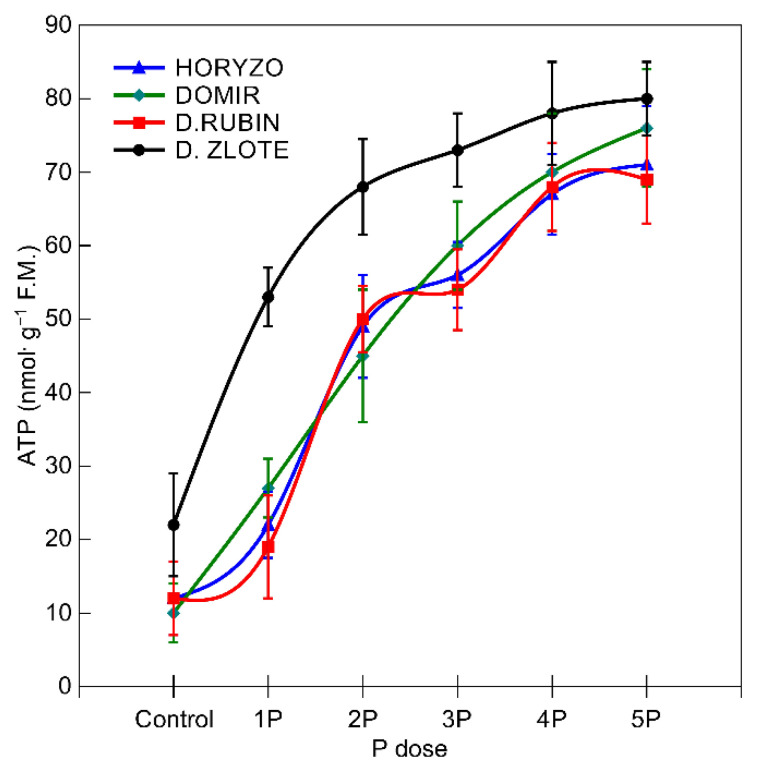
Changes in the intracellular concentration of adenosine triphosphate in roots of rye varieties grown on the post-flotation sediment under variable P supplementation. The fresh root tissue was homogenised in 4.5% perchloric acid and purified, and the ATP content was determined fluorometrically by glycerol phosphorylation at λ_ex_ = 535 nm and λ_em_ = 587 nm. Data represent the means of *n* = 9 replications, and vertical bars represent 95% confidence interval after a post hoc Tukey HSD test (interaction P dose × variety), with the letters denoting homogeneous groups being omitted for a clear view.

The abovementioned mechanisms involved in P mobilisation are strictly dependent on the metabolic energy supply [65]. The major sites of ATP synthesis in plants are the ATP synthase protein complexes driven by the proton motive force established across the thylakoid membranes in chloroplasts and the inner membrane of the mitochondria [79]. The ATP concentrations determined in the current experiment in the root tissue of the control plants (0P) were statistically higher in Dankowskie Zlote in comparison to Dankowskie Rubin, Horyzo, and Domir (Figure 5). Supplementation of the plants with increasing amounts of P resulted in a significant increase in the intracellular ATP level. This was most substantial in Dankowskie Zlote, reaching 85% of the maximum ATP content already at the 2P dose. In the other rye varieties, the observed increase in ATP concentration in response to higher P supply was not so well defined. The levels of ATP accumulation in the roots of rye, resulting from the ability to both synthesise and economically manage ATP resources, are consistent with the activity levels of the two P-mobilising processes characterised in this paper. Among the examined rye varieties, Dankowskie Zlote seems to be better equipped with mechanisms coordinating the generation of adequate ATP and is able to use the ATP pool more effectively. Importantly, the root-based respiratory processes involved in ATP synthesis consume 40% of the daily photosynthetically fixed carbon [71].

### 3.3. The Photosynthetic Performance Is Essential in Determining the Capacity of Rye for P Mobilisation

The growth and development of plants are ultimately driven by light energy captured through the process of photosynthesis, which is the basis for plant productivity and survival strategies under adverse conditions. At the same time, photosynthesis is well established to be the primary target of many forms of environmental stress, including lack of nutrients [35]. P is an essential element in compounds such as ATP, NADPH, and sugar phosphates; therefore, even marginal P deficiency may have a major impact on photosynthesis [80].

**Figure 6:**
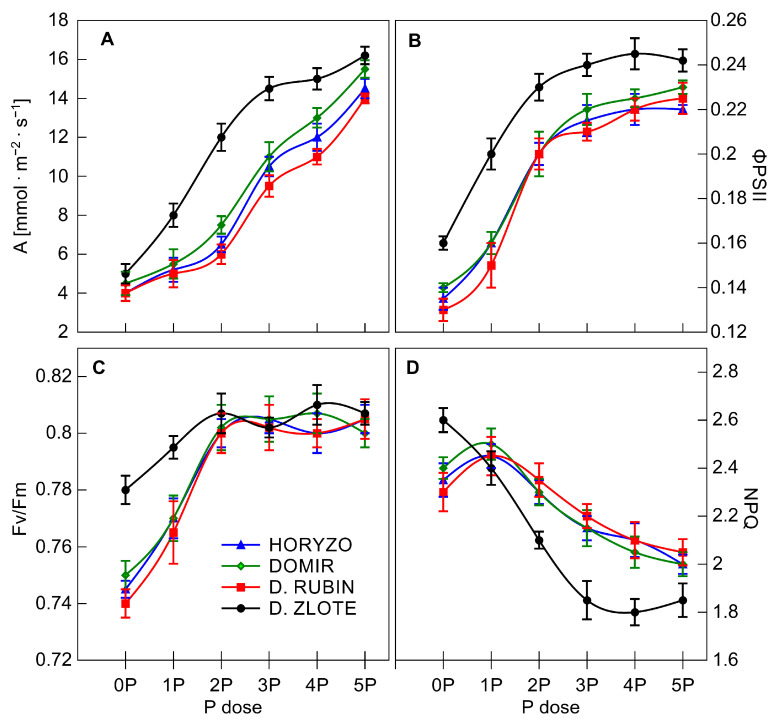
Photosynthetic parameters in rye varieties grown on the post-flotation sediment under variable P supplementation. The intact leaves of dark-adapted plants were illuminated with a 1.2 s pulse of saturating light (PPFD 9500 μmol·m^2^·s^−1^) at 25 °C in the presence of 415 μmol·mol^−1^ CO_2_, followed by the switch on of actinic light (PPFD 850 μmol·m^2^·s^−1^) for 30 min to reach a steady state. The CO_2_ assimilation rate, *A* (**A**), photochemical efficiency, *ΦPSII* (**B**), maximum quantum efficiency, *Fv/Fm* (**C**), and nonphotochemical quenching, *NPQ* (**D**) were determined. Data represent the means of *n* = 9 replications, and vertical bars represent 95% confidence interval after a post hoc Tukey HSD test (interaction P dose × variety), with the letters denoting homogeneous groups being omitted for a clear view.

In our study, the P limitation resulted in significant inhibition of the photosynthetic carbon fixation in leaves of the rye varieties grown on the post-flotation sediment. The harmful effect on CO_2_ assimilation, measured in intact leaves, was greater in Dankowskie Rubin, Horyzo, and Domir, as compared to Dankowskie Zlote (Figure 6A). However, even in control plants (0P) challenged with severe P deficiency, carbon assimilation was strongly reduced but not terminated, in line with previous reports [81]. The CO_2_ assimilation rate increased with increasing P supply, with this being induced rapidly in leaves of Dankowskie Zlote upon exposure to the 1P level of supplementation and developed further to reach 91% of the maximum rate already at the 3P dose. In contrast, in the other varieties, there was no such fast induction observed. The inhibitory effect of P deficiency on the photosynthetic carbon fixation might be explained in a number of ways. A low level of P in leaf mesophyll cells may have a direct effect on photosynthesis through depressed Pi availability in the chloroplast [80]. P starvation affects CO_2_ assimilation by reducing the ATP-dependent regeneration of ribulose-1,5-bisphosphate and the abundance and activity of the key enzymes of the Calvin cycle, including Rubisco and Rubisco activase [81,82]. In addition, inhibition of photosynthesis may result from decreased activity of the photosynthetic electron transport chain (ETC) in thylakoid membranes [35,83].

Measurements of chlorophyll fluorescence were used here to detail functioning of ETC in intact leaves. Among various components of photosynthetic apparatus, the PSII complex is particularly sensitive to environmental stress [84]. Indeed, a considerable decrease in the activity of PSII was observed in leaves of the studied rye varieties in response to P deficiency. The parameter ΦPSII provides an estimate of the actual photochemical efficiency of PSII [85], and, as such, it can give a measure of the rate of linear electron transport. The decrease in CO_2_ fixation observed in rye was accompanied by inhibition of the electron transport through PSII, as indicated by the fall in ΦPSII; however, this inhibition was less marked in Dankowskie Zlote (Figure 6B). This fall implies that P deficiency provoked a significant reduction in the photosynthetic conversion of absorbed light energy in PSII and decreased transfer effectiveness through the ETC in rye varieties grown under very low P [35,86]. Increasing amounts of P resulted in a progressive increase in ΦPSII, reflecting a significant improvement in the linear electron flow and overall photosynthesis.

Fv/Fm, a measure of the maximum (or intrinsic) efficiency of PSII, was close to the value of 0.8 in plants well fed with P (Figure 6C). Exposure of rye varieties to suboptimal P levels, in contrast to ΦPSII, did not have any instant effect on Fv/Fm. Throughout the experiment, the maximum efficiency of PSII declined significantly only in Dankowskie Rubin, Horyzo, and Domir exposed to 0P and 1P levels of P. At the same time, there was only a marginal reduction in this parameter in Dankowskie Zlote grown under control conditions (0P). The decrease in Fv/Fm observed in response to severe P deficiency clearly reflects the occurrence of photoinhibition in the PSII reaction centres due to an over-reduction of the photosynthetic ETC and an accumulation of the damaged photosystems in chloroplast [35,83]. Under normal conditions, photosynthetic organisms are able to overcome photodamage via the rapid and efficient repair of PSII; however, the rate of repair may be severely depressed under stress conditions [84]. The repair process of photodamaged PSII is regulated through the reversible phosphorylation of the core proteins catalysed by protein kinases (STN8 and STN7) and the photosystem II core phosphatase (PBCP). Thus, it seems possible that the episodes of photoinhibition observed in leaves of rye varieties are a consequence of the disturbance in the phosphorylation–dephosphorylation process due to depressed Pi and ATP availability in chloroplast. In contrast, the lack of substantial changes in Fv/Fm in Dankowskie Zlote, as well as in the other rye varieties when subjected to the 2P–5P supplementation doses (Figure 6C), indicates that the reduction in ΦPSII in these combinations (Figure 6B) resulted from a downregulation of the linear electron flow, as opposed to damage to PSII [35,87]. This downregulation is a mechanism to match the lower demand for NADPH in the dark reactions of the Calvin cycle when CO_2_ assimilation is inhibited. Simultaneously, it allows avoiding the generation of reactive oxygen species at the level of PSII and PSI, when availability of NADP^+^ is reduced due to Pi deficiency [88].

It has been widely observed that, under stress conditions, plants increase the dissipation of absorbed light energy in the form of heat, measured as nonphotochemical quenching of chlorophyll fluorescence (NPQ; [86]). Indeed, P limitation resulted in a substantial increase in NPQ in leaves of rye varieties grown on the post-flotation sediment (Figure 6D), reversibly correlated with downregulation of the linear electron transport (Figure 6B). The increasing P application into the post-flotation waste gradually diminished the level of nonphotochemical quenching in all varieties. The observed increase in NPQ could be explained by changes in activity of the chloroplast ATP synthase, driven by the proton motive force (ΔpH) established across the thylakoid membrane [79]. P deficiency may reduce the ADP and Pi concentration in leaf mesophyll and, thus, in the chloroplast to a level inhibiting the activity of the ATP synthase. As a consequence, protons accumulate in the thylakoid, provoking lumen acidification, which in turn triggers enhanced energy dissipation in nonphotochemical processes [80]. Importantly, increased dissipation of energy as heat protects PSII against photodamage. The NPQ values determined in the rye varieties imply that Dankowskie Zlote is better equipped with mechanisms protecting against photoinhibition, which is in agreement with the higher level of Fv/Fm measured in control plants (Figure 6C).

Overall, the responses of photosynthesis in all rye varieties were very much in line with the responses of the physiological processes involved in P mobilisation observed under variable P availability. As demonstrated, better functioning of the photosynthetic electron transport chain and higher performance of the photosynthetic carbon fixation in leaves were clearly linked with higher activity of the PM H^+^-ATPase and higher exudation rates in roots.

### 3.4. Effectiveness of the P-Mobilising Mechanisms in the Context of P Rendered Available in the Soil Solution

**Figure 7:**
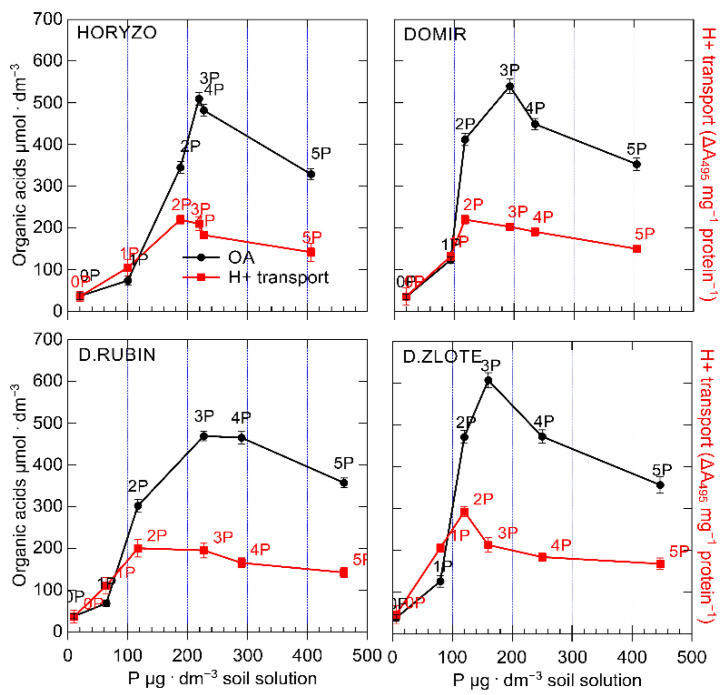
The level of OA exudation and proton efflux in roots of rye varieties grown on the post-flotation sediment in relation to the P concentration in soil solution. Plants were subjected to 0 (0P), 22 (1P), 44 (2P), 66 (3P), 88 (4P), and 110 (5P) mg P·kg^−1^. Soil solutions were collected, and the plasma membrane vesicles were isolated as described in Figure 3 and Figure 4, respectively. Data represent the means of *n* = 9 replications ± SE.

The earlier induction and the higher activities of the H^+^-ATPase-mediated proton efflux and the OA exudation per available P unit in the rhizosphere of Dankowskie Zlote (Figure 7), in contrast to Dankowskie Rubin, Horyzo, and Domir, might reflect better adaptations to low-P conditions in older, more primitive rye cultivars. This is further supported by more economical utilisation of the ATP pool in proton transport in this variety (Figure 4). The selection and breeding of rye varieties towards higher yields obtained under optimal conditions, i.e., on P-rich or well fertilised soils, may lead to partial loss of such characteristics. The processes of breeding inevitably contribute to a reduction in genetic diversity, with an increased susceptibility to abiotic and biotic stress being the consequence of such a genetic erosion [28]. Therefore, we suggest that the varieties with a more primitive genetic background might be potentially more suitable for growth and survival on the post-flotation sediments.

The observed dynamics and the shift in appearance of the maximum activities of both processes involved in P mobilisation indicate that the PM H^+^-ATPase-driven acidification is a mechanism dominating at the lower P levels in soil solution. The highest H^+^ efflux and the highest OA exudation rates occurred at the 2P and 3P combination, respectively (Figure 7). Thus, at the lower P availability, ATP is being rather used for H^+^-coupled hydrolysis instead of energy-intensive synthesis of the OA and the carboxylate protein transporters.

The P concentration in soil solution is the resultant of two parallel processes—P mobilisation in the rhizosphere and its extraction by roots. Thus, the lower P contents in soil solution, at which high activities of the H^+^ efflux and the OA exudation were observed in Dankowskie Zlote, reflect not only the high effectiveness of these mechanisms, but also improved P acquisition from soil, which is consistent with the role of the H^+^ gradient generated across plasma membrane in activation of the high-affinity phosphate transporters PHT1 [77].

**Figure 8:**
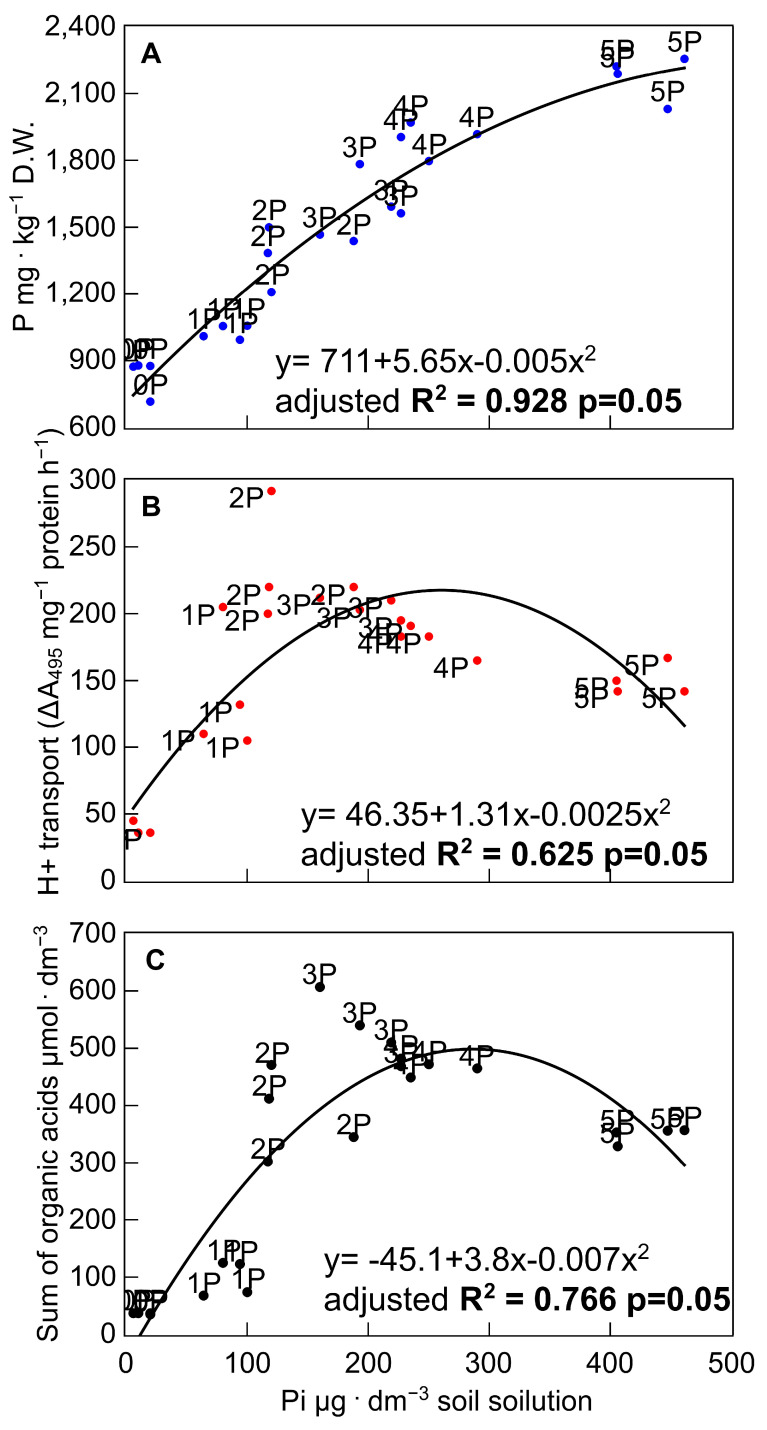
Correlation analysis between P concentration in shoots (**A**), H^+^-ATPase-driven proton efflux (**B**), and OA exudation (**C**) and the P concentration in soil solution from pots in which rye varieties were grown on the post-flotation sediment under 0 (0P), 22 (1P), 44 (2P), 66 (3P), 88 (4P), and 110 (5P) mg P·kg^−1^. The coefficients of determination (*R*^2^) and curve fitting were determined using the data from all treatments (*p* < 0.05) with QtiPlot 1.0.0-rc13 software.

The improved P availability in the soil solution promoted an increased accumulation of P in the tissues of all tested rye varieties (Figure 8A). The soil solution P content and the intracellular P concentration were strongly correlated, with *R*^2^ = 0.928. Importantly, a value in excess of 0.25 mg·dm^−3^ (4P combination) is required to ensure that the tissue concentration is reported as optimal [49,59]. Furthermore, a significant relationship was found between the phosphate concentration in soil solution and H^+^ transport (*R*^2^ = 0.625; Figure 8B) and the sum of OA exuded (*R*^2^ = 0.766; Figure 8C). The lower correlation and *R*^2^ value in the case of the PM H^+^-ATPase-mediated proton transport, in contrast to the OA exudation, most probably result from the parallel involvement of this mechanism in the multiple P-acquisition processes. These include rhizosphere acidification, the release of organic anions, and the energisation of phosphate transporters in the plasma membrane [61,65,74].

## 4. Conclusions

On the basis of the results presented in this work, the high total Cu contents found in the post-flotation tailings seem not to be the most critical factor limiting growth and survival on this type of waste. It is rather the low bioavailability of nutrients, particularly P, resulting from the physicochemical properties of the substrate, i.e., alkaline/calcareous characteristics. Therefore, the selection of plant species and genotypes equipped in morphological, physiological, and biochemical adaptation to overcome the low availability and poor mobility of P is of major importance.

We clearly demonstrated the relationship between P availability in the post-flotation sediment and the activity of the mechanisms involved in P mobilisation in rye varieties. Both the proton efflux and the OA exudation in roots were reversibly correlated with the level of available P in the rhizosphere. Importantly, we showed that there is a minimum threshold level of P in the soil solution, below which the proton release and the exudation into the root zone are not enhanced. Furthermore, PM H^+^-ATPase-mediated proton transport seems to be a predominant mechanism at lower P levels; with increasing P concentration in the soil solution, an increased contribution of the carboxylic acids in P mobilisation was observed.

We suggest that the older rye varieties, carrying more primitive genetic characteristics, are better equipped with the molecular mechanisms essential for survival strategies in low-P and contaminated environments and, as such, might be more suitable for growth on the post-flotation copper tailings. Dankowskie Zlote accumulated more ATP from the available P pool, as well as demonstrated higher activity of PM H^+^-ATPase, more effective H^+^ transport per ATP unit, and more efficient exudation of carboxylates. The high activities of the mechanisms involved in P mobilisation in Dankowskie Zlote are determined, at least partially, by the high efficiency of photosynthetic electron transport and photosynthetic CO_2_ fixation observed in this variety.

To recapitulate, rye as a species, particularly the more primitive genotypes, seems potentially promising for use in revitalisation of the post-flotation tailings; however, this has to be further supported by long-term in situ research. The results presented here will certainly inform future works in developing strategies for successful revitalisation of degraded lands.

## Figures and Tables

**Table 1:** Total content of selected elements in the post-flotation sediment.

SiO_2_	Ca	Mg	K	P	Fe	Mn	Zn	Cu
% DM					mg·kg^−1^ (DM)			
54 ± 1.8	16.3 ± 0.5	2.4 ± 0.1	2.7 ± 0.1	0.08 ± 0.005	18,000 ± 80	1900 ± 9	70 ± 9	1800 ± 90

**Table 2:** Granulometric composition of the post-flotation sediment (% DM).

Granulometric Class			
**>2.0 mm**	**0.05–2.0 mm**	**0.002–0.05 mm**	**<0.002 mm**
0	1–13	63–85	5–35

## Data Availability

The data presented in this study are available within the article.

## References

[1] Sverdrup H.U., Olafsdottir H.A., Ragnarsdottir K.V. (2019). On the long-term sustainability of copper, zinc and lead supply, using a system dynamics model. Res. Cons. Rec. X.

[2] Younger P.L., Wolkersdorfer C. (2004). Mining Impacts on the Fresh Water Environment: Technical and Managerial Guidelines for Catchment Scale Management. Mine Water Environ..

[3] Edraki M., Baumgartl T., Manlapig E., Bradshaw D., Franks D.M., Moran C.J. (2014). Designing mine tailings for better environmental, social and economic outcomes: A review of alternative approaches. J. Clean. Prod..

[4] Kossoff D., Dubbin W.E., Alfredsson M., Edwards S.J., Mackline M.G., Hudson-Edwards K.A. (2014). Mine tailings dams: Characteristics, failure, environmental impacts, and remediation. Appl. Geochem..

[5] Li X., Huang L. (2014). Toward a New Paradigm for Tailings Phytostabilization—Nature of the Substrates, Amendment Options, and Anthropogenic Pedogenesis. Crit. Rev. Environ. Sci. Technol..

[6] Mason L., Prior T., Mudd G., Giurcoa D. (2011). Availability, addiction and alternatives: Three criteria for assessing the impact of peak minerals on society. J. Clean. Prod..

[7] Onuaguluchi O., Eren Ö. (2012). Recycling of copper tailings as an additivein cement mortars. Constr. Build. Mater..

[8] Lottermoser B.G. (2010). Mine Wastes, Characterization, Treatment and Environmental Impacts.

[9] Asensio V., Vega F.A., Singh B.R., Covelo E.F. (2013). Effects of tree vegetation and waste amendments on the fractionation of Cr, Cu, Ni, Pb and Zn in polluted mine soils. Sci. Total Environ..

[10] Tshibangu M.I., Nsahlai V.I., Kiatoko M.H., Hornick J.L. (2014). Heavy Metals Concentration in Adenodolichos rhomboideus (O. Hoffm.) Harms. Forage Growing on Mining Tailings in South East of Democratic Republic of Congo: Influence of Washing, pH and Soil Concentrations. Int. J. Curr. Res. Biosci. Plant Biol..

[11] Baycu G., Tolunay D., Ozden H., Csatari I., Karadag S., Agba T., Rognes S.E. (2015). An abandoned copper mining site in cyprus and assessment of metal concentrations in plants and soil. Int. J. Phytoremediat..

[12] Carrasco L., Caravaca F., Azcón R., Roldán A. (2009). Soil acidity determines the effectiveness of an organic amendment and a native bacterium for increasing soil stabilisation in semiarid mine tailings. Chemosphere.

[13] Sheoran V., Sheoran A.S., Poonia P. (2010). Soil Reclamation of Abandoned Mine Land by Revegetation. Int. J. Soil Sediment Water.

[14] Lilić J., Cupać S., Lalević B., Andrić V., Gajić-Kvaščev M. (2014). Pedological characteristics of open-pit Cu wastes and postflotation tailings (Bor, Serbia). J. Soil Sci. Plant Nutr..

[15] Wang L., Ji B., Hu Y., Liu R., Sun W. (2017). A review on in situ phytoremediation of mine tailings. Chemosphere.

[16] Chen B., Tang X., Zhu Y., Christie P. (2005). Metal concentrations and mycorrhizal status of plants colonizing copper mine tailings; potential for revegetation. Sci. China.

[17] Chen B.D., Zhu Y.G., Duan J., Xiao X.Y., Smith S.E. (2007). Effects of the arbuscular mycorrhizal fungus Glomus mosseae on growth and metal uptake by four plant species in copper mine tailings. Environ. Pollut..

[18] Chaturvedi N., Ahmed M.J., Dhal N.K. (2013). Effects of iron ore tailings on growth and physiological activities of *Tagetes patula* L.. J. Soils Sed..

[19] Chaignon V., Hinsinger P. (2003). A Biotest for Evaluating Copper Bioavailability to Plants in a Contaminated Soil. J. Environ. Qual..

[20] Spiak Z., Gediga K. (2012a). Assessment of the applicability of some mineral wastes for revitalization of a postflotation dumping site. Przem. Chem..

[21] Tordoff G.M., Baker A.J.M., Willis A.J. (2000). Current approaches to the revegetation and reclamation of metalliferous mine wastes. Chemosphere.

[22] Spiak Z., Gediga K. (2012b). Applicability of selected plant species for the occupance of land degraded by copper industry. Przem. Chem..

[23] Spiak Z., Gediga K. (2009). Usefulness of selected mineral wastes for reclamation of copper industry dumping site. Environ. Prot. Eng..

[24] Gajić G., Djurdjević L., Kostić O., Jarić S., Mitrović M., Pavlović P. (2018). Ecological Potential of Plants for Phytoremediation and Ecorestoration of Fly Ash Deposits and Mine Wastes. Front. Environ. Sci..

[25] Schroeder K., Rufaut C.G., Smith C., Mains D., Craw D. (2005). Rapid plant-cover establishment on gold mine tailings in southern New Zealand: Glasshouse screening trials. Int. J. Phytoremediat..

[26] Mains D., Craw D., Rufaut C.G., Smith C.M. (2006). Phytostabilization of gold mine tailings, New Zealand. Part 1, Plant establishment in alkaline saline substrate. Int. J. Phytoremediat..

[27] Schlegel R.H.J. (2014). Rye: Genetics, Breeding, and Cultivation.

[28] Targońska M., Bolibok-Brągoszewska H., Rakoczy-Trojanowska M. (2015). Assessment of Genetic Diversity in Secale cereale Based on SSR Markers. Plant. Mol. Biol. Rep..

[29] Mench M., Lepp N., Bert V., Schwitzguébel J.-P., Gawronski S.W., Schröder P., Vangronsveld J. (2010). Successes and limitations of phytotechnologies at field scale: Outcomes, assessment and outlook from COST Action 859. J. Soils Sed..

[30] Burges A., Alkorta I., Epelde L., Garbisu C. (2018). From phytoremediation of soil contaminants to phytomanagement of ecosystem services in metal contaminated sites. Int. J. Phytoremediat..

[31] Kidd P., Mench M., Álvarez-López V., Bert V., Dimitriou I., Friesl-Hanl W., Puschenreiter M. (2015). Agronomic practices for improving gentle remediation of trace element-contaminated soils. Int. J. Phytoremed..

[32] Schachtschabel P. (1954). Das pflanzenverfügbare Magnesium des Boden und seine Bestimmung. Zeitschift Pflanz. Düngung Bodenkd..

[33] Egner H., Riehm H., Methodenbuch I., von Thun R., Hermann R., Knickmann E. (1955). Die Doppellactatmethode, zit.

[34] Lancashire P.D., Bleiholder H., Van Den Boom T., Langeluddeke P., Stauss R., Weber E., Witzenberger A. (1991). A uniform decimal code for growth stages of crops and weeds. Ann. Appl. Biol..

[35] Stepien P., Johnson G.N. (2009). Contrasting responses of photosynthesis to salt stress in the glycophyte Arabidopsis thaliana and the halophyte Thellungiella halophila. Role of the plastid terminal oxidase as an alternative electron sink. Plant Physiol..

[36] Lund A., Fuglsang A.T. (2012). Purification of plant plasma membranes by two-phase partitioning and measurement of H+ pumping. Met. Mol. Biol..

[37] Johansson F., Olbe M., Sommarin M., Larsson C. (1995). Brij 58, a polyoxyethylene acyl ether, creates membrane vesicles of uniform sidedness. A new tool to obtain inside-out (cytoplasmic side-out) plasma membrane vesicles. Plant J..

[38] Gallahger S.R., Leonard R.T. (1982). Effect of vanadate, molybdate and azide on membrane associated ATPase and soluble phosphatase activities of corn roots. Plant Physiol..

[39] Sze H. (1985). H+-translocating ATPase. Advances using membrane vesicles. Ann. Rev. Plant Physiol..

[40] Ames B.N. (1966). Assay of inorganic phosphate, total phosphate and phosphatases. Meth. Enzymol..

[41] Kłobus G., Buczek J. (1995). The role of plasma membrane oxidoreductase activity in proton transport. J. Plant Physiol..

[42] Murphy J., Riley J.P. (1962). A modified single solution method for the determination of phosphate in natural waters. Anal. Chim. Acta.

[43] Watanabe F.S., Olsen S.R. (1965). Test of an ascorbic acid method for determining phosphorus in water and NaHCO3 extracts from soil. Soil Sci. Am. Proc..

[44] Kasowska D., Gediga K., Spiak Z. (2018). Heavy metal and nutrient uptake in plants colonizing post-flotation copper tailings. Environ. Sci. Pollut. Res. Int..

[45] Kabata-Pendias A., Pendias H. (2001). Trace Elements in Soils and Plants.

[46] Bradl H.B. (2005). Heavy Metals in the Environment: Origin, Interaction and Remediation.

[47] Mengel K., Kirkby E.A., Kosegarten H., Appel T. (2001). Copper. Principles of Plant Nutrition.

[48] Hock B., Elstner F.E. (2005). Plant Toxicology.

[49] Jones J.B. (2012). Plant Nutrition and Soil Fertility Manual.

[50] Ponizovsky A.A., Allen H.E., Ackerman A.J. (2007). Copper activity in soil solutions of calcareous soils. Environ. Pollut..

[51] Elbana T.A., Selim H.M. (2011). Copper Mobility in Acidic and Alkaline Soils: Miscible Displacement Experiments. Soil Sci. Soc. Am. J..

[52] Haynes W.M., Lide D.R., Bruno T.J. (2016). CRC Handbook of Chemistry and Physics: A Ready-Reference Book of Chemical and Physical Data.

[53] Reed S.C., Yang X., Thornton P.E. (2015). Incorporating phosphorus cycling into global modeling efforts: A worthwhile, tractable endeavor. New Phytol..

[54] Tiessen H., White P.J., Hammond J.P. (2008). Phosphorus in the global environment. The Ecophysiology of Plant-Phosphorus Interactions.

[55] Lis J., Pasieczna A., Mojski J.E., Przeniosło S., Sylwestrzak H., Strzelecki R., Wołkowicz S. (2012). Geochemical Atlas of Poland.

[56] Salminen R., Batista M.J., Bidovec M., Demetriades A., De Vivo B., De Vos W., Durism M., Gilucis A., Gregorauskiene V., Halamic J. (2005). Geochemical Atlas of Europe. Part 1—Background Information, Methodology and Maps.

[57] Kęsik K., Jadczyszyn T., Lipiński W., Jurga B. (2015). Adaptation of the Mehlich 3 procedure for routine determination of phosphorus, potassium and magnesium in soil. Przem. Chem..

[58] Hawkesford M., Horst W., Kichey T., Lambers H., Schjoerring J., Møller I.S., White P., Marschner P. (2012). Chapter 6—Functions of Macronutrients. Marschner’s Mineral Nutrition of Higher Plants.

[59] Campbell C.R. Reference Sufficiency Ranges for Plant Analysis in the Southern Region of the United States.

[60] Lambers H., Shane M.W., Cramer M.D., Pearse S.J., Veneklaas E.J. (2006). Root Structure and Functioning for Efficient Acquisition of Phosphorus: Matching Morphological and Physiological Traits. Ann. Bot..

[61] Lambers H., Clode P., Hawkins H.J., Laliberté E., Oliveira R.S., Reddell P., Shane M.W., Stitt M., Weston P., Plaxton W.C., Lambers H. (2015). Metabolic adaptations of the non-mycotrophic Proteaceae to soils with low phosphorus availability. Annual Plant Reviews.

[62] Long M.H., McGlathery K.J., Zieman J.C., Berg P. (2008). The role of organic acid exudates in liberating phosphorus from seagrass-vegetated carbonate sediments. Limnol. Oceanogr..

[63] Ryan P., Delhaize E., Jones D. (2001). Function And Mechanism Of Organic Anion Exudation From Plant Roots. Annu. Rev. Plant Physiol. Plant Mol. Biol..

[64] Chen Y.L., Dunbabin V.M., Diggle A.J., Siddique K.H.M., Rengel Z. (2013). Phosphorus starvation boosts carboxylate secretion in P-deficient genotypes of Lupinus angustifolius with contrasting root structure. Crop Pasture Sci..

[65] Canarini A., Kaiser C., Merchant A., Richter A., Wanek W. (2019). Root exudation of primary metabolites: Mechanisms and their roles in plant responses to environmental stimuli. Front. Plant Sci..

[66] Roelofs R.F.R., Rengel Z., Cawthray G.R., Dixon K.W., Lambers H. (2001). Exudation of carboxylates in Australian Proteaceae: Chemical composition. Plant Cell Environ..

[67] Veneklaas E.J., Stevens J., Cawthray G.R., Turner S., Grigg A.M., Lambers H. (2003). Chickpea and white lupin rhizosphere carboxylates vary with soil properties and enhance phosphorus uptake. Plant Soil.

[68] White P.J., Hammond J.P., White P.J., Hammond J.P. (2008). Phosphorus nutrition of terrestrial plants. The Ecophysiology of Plant-Phosphorus Interactions.

[69] Hunter P.J., Teakle G.R., Bending G.D. (2014). Root traits and microbial community interactions in relation to phosphorus availability and acquisition, with particular reference to Brassica. Front. Plant Sci..

[70] Hinsinger P., Plassard C., Tang C.X., Jaillard B. (2003). Origins of root-mediated pH changes in the rhizosphere and their responses to environmental constraints: A review. Plant Soil.

[71] Uhde-Stone C., Sulieman R.S., Lam-Son P.T. (2017). White Lupin: A Model System for Understanding Plant Adaptation to Low Phosphorus Availability. Legume Nitrogen Fixation in Soils with Low Phosphorus Availability.

[72] Howell T., Moriconi J.I., Zhao X., Hegarty J., Fahima T., Santa-Maria G.E., Dubcovsky J. (2019). A wheat/rye polymorphism affects seminal root length and yield across different irrigation regimes. J. Exp. Bot..

[73] Hinsinger P., Herrmann L., Lesueur D., Robin A., Trap J., Waithaisong K., Plassard C., Plaxton W.C., Lambers H. (2015). Impact of roots, microorganisms and microfauna on the fate of soil phosphorus in the rhizosphere. Annual Plant Reviews.

[74] Yu W., Kan Q., Zhang J., Zeng B., Chen Q. (2016). Role of the plasma membrane H+-ATPase in the regulation of organic acid exudation under aluminum toxicity and phosphorus deficiency. Plant Signal. Behav..

[75] Haruta M., Gray W.M., Sussman M.R. (2015). Regulation of the plasma membrane proton pump (H^+^-ATPase) by phosphorylation. Curr. Opin. Plant Biol..

[76] Kerkeb L., Venema K., Donaire J., Rodriguez-Rozales M. (2002). Enhanced H+/ATP coupling ratio of H+-ATPase and increased 14-3-3 protein content in plasma membrane of tomato cells upon osmotic shock. Physiol. Plant.

[77] Poirier Y., Jung J.-Y., Plaxton W.C., Lambers H. (2015). Phosphate transporters. Annual Plant Reviews.

[78] Zhu Y., Yan F., Zörb C., Schubert S. (2005). Link Between Citrate and Proton Release by Proteoid Roots of White Lupin (*Lupinus albus* L.) Grown Under Phosphorus-deficient Conditions?. Plant Cell Physiol..

[79] De Col V., Fuchs P., Nietzel T., Elsässer M., Voon C.P., Candeo A., Seeliger I., Fricker M.D., Grefen C., Møller I.M. (2017). ATP sensing in living plant cells reveals tissue gradients and stress dynamics of energy physiology. eLife.

[80] Carstensen A., Herdean A., Schmidt S.B., Sharma A., Spetea C., Pribil M., Husted S. (2018). The impacts of phosphorus deficiency on the photosynthetic electron transport chain. Plant Physiol..

[81] Hammond J.P., White P.J. (2008). Sucrose transport in the phloem: Integrating root responses to phosphorus starvation. J. Exp. Bot..

[82] Chu S., Li H., Zhang X., Yu K., Chao M., Han S., Zhang D. (2018). Physiological and proteomics analyses reveal low-phosphorus stress affected the regulation of photosynthesis in soybean. Int. J. Mol. Sci..

[83] Stepien P., Johnson G.N. (2018). Plastid terminal oxidase requires translocation to the grana stacks to act as a sink for electron transport. Proc. Natl. Acad. Sci. USA.

[84] Murata N., Takahashi S., Nishiyama Y., Allakhverdiev S.I. (2007). Photoinhibition of photosystem II under environmental stress. Biochim. Biophys. Acta.

[85] Genty B., Briantain J.M., Baker N.R. (1989). The relationship between the quantum yield of photosynthetic electron transport and quenching of chlorophyll fluorescence. Biochim. Biophys. Acta.

[86] Maxwell K., Johnson G.N. (2000). Chlorophyll fluorescence—A practical guide. J. Exp. Bot..

[87] Golding A.J., Johnson G.N. (2003). Down-regulation of linear and activation of cyclic electron transport during drought. Planta.

[88] Asada K. (2006). Production and scavenging of reactive oxygen species in chloroplasts and their functions. Plant Physiol..

